# Biodegradable and Biocompatible Adhesives for the Effective Stabilisation, Repair and Regeneration of Bone

**DOI:** 10.3390/bioengineering9060250

**Published:** 2022-06-10

**Authors:** Antzela Tzagiollari, Helen O. McCarthy, Tanya J. Levingstone, Nicholas J. Dunne

**Affiliations:** 1 School of Mechanical and Manufacturing Engineering, Dublin City University, D09 NA55 Dublin, Ireland; antzela.tzagiollari2@mail.dcu.ie (A.T.); tanya.levingstone@dcu.ie (T.J.L.); 2 Centre for Medical Engineering Research, Dublin City University, D09 NA55 Dublin, Ireland; 3 School of Pharmacy, Queen’s University, Belfast BT9 7BL, UK; h.mccarthy@qub.ac.uk; 4 School of Chemical Sciences, Dublin City University, D09 NA55 Dublin, Ireland; 5 Biodesign Europe, Dublin City University, D09 NA55 Dublin, Ireland; 6 Tissue, Engineering Research Group, Department of Anatomy and Regenerative Medicine, Royal College of Surgeons in Ireland, D02 PN40 Dublin, Ireland; 7 Advanced Manufacturing Research Centre (I-Form), School of Mechanical and Manufacturing Engineering, Dublin City University, D09 NA55 Dublin, Ireland; 8 Advanced Processing Technology Research Centre, Dublin City University, D09 NA55 Dublin, Ireland; 9 Trinity Centre for Biomedical Engineering, Trinity Biomedical Sciences Institute, Trinity College Dublin, D02 PN40 Dublin, Ireland; 10 Advanced Materials and Bioengineering Research Centre (AMBER), Trinity College Dublin, D02 PN40 Dublin, Ireland; 11 Department of Mechanical and Manufacturing Engineering, School of Engineering, Trinity College Dublin, D02 PN40 Dublin, Ireland

**Keywords:** bone fractures, bioadhesives, bone repairing, biomimetic adhesives

## Abstract

Bone defects and complex fractures present significant challenges for orthopaedic surgeons. Current surgical procedures involve the reconstruction and mechanical stabilisation of complex fractures using metal hardware (i.e., wires, plates and screws). However, these procedures often result in poor healing. An injectable, biocompatible, biodegradable bone adhesive that could glue bone fragments back together would present a highly attractive solution. A bone adhesive that meets the many clinical requirements for such an application has yet to be developed. While synthetic and biological polymer-based adhesives (e.g., cyanoacrylates, PMMA, fibrin, etc.) have been used effectively as bone void fillers, these materials lack biomechanical integrity and demonstrate poor injectability, which limits the clinical effectiveness and potential for minimally invasive delivery. This systematic review summarises conventional approaches and recent developments in the area of bone adhesives for orthopaedic applications. The required properties for successful bone repair adhesives, which include suitable injectability, setting characteristics, mechanical properties, biocompatibility and an ability to promote new bone formation, are highlighted. Finally, the potential to achieve repair of challenging bone voids and fractures as well as the potential of new bioinspired adhesives and the future directions relating to their clinical development are discussed.

## 1. Introduction

Bone fractures are common injuries resulting from trauma or diseases such as osteoporosis and bone cancer [1]. A patient’s age, gender, lifestyle and pre-existing medical conditions are all important factors affecting the risk of a fracture occurring and the likelihood that complications will occur during the repair process [2,3]. Overall, according to a Global Burden of Disease study, an estimated 178 million individuals (53% males and 47% females) worldwide suffered bone fractures in 2019, leading to an increase of approximately 34% since 1990 [4].

**Figure 1:**
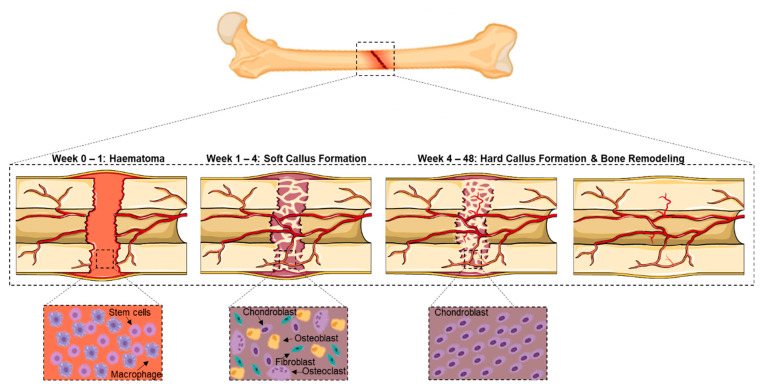
Stages of bone healing: (1) haematoma formation from stem and macrophage cells at the fracture site (week 0–1), (2) soft callus formation at the fracture site, from chondroblast, osteoblast, fibroblast and osteoclast, replaces the hematoma (week 1–4) and (3) hard callus replaces the soft callus, using chondroblast cells and, after week 6–8, bone starts to replace the hard callus (week 4–48).

During the normal bone fracture healing process, three overlapping stages occur: (1) inflammation, (2) bone production and (3) bone remodelling (Figure 1). Initial bleeding into the fracture area is followed by inflammation and clotting of blood at the fracture site. These processes involve haematopoietic and immune cells within the bone marrow and mesenchymal stem cells (MSCs) from the surrounding tissue and bone marrow [5,6]. Clotted blood is replaced with fibrous tissue and cartilage (soft callus) within 2 to 4 weeks. Callus formation around the fractured bone provides early stabilisation and protects the repair tissue from external forces [7]. Subsequently, the calcium formation that is laid down in the matrix within the next 4 to 12 months results in the callus becoming visible on radiographic images. The successful restoration of the original shape and structure of bone (i.e., bone remodelling) is the final stage in the normal healing process. In some incidences, bone healing does not occur in accordance with the normal bone repair processes. For example, micromotion at the repair site can interrupt the healing process and lead to other possible complications, such as bleeding into a joint space that causes the joint to swell (haemarthrosis) and blood clot formation that can cause blockage within a blood vessel, locally or elsewhere in the body. Non-union fractures occur when the broken bones are not able to heal due to insufficient nutrition, limited blood supply or inadequate stability (poor immobilisation). In many cases, the healing process can last from months to years [8]. 

Current surgical procedures for the treatment of bone fractures involve the use of invasive techniques for the reconstruction and mechanical stabilisation of the fractures using metal hardware (e.g., wires, screws, pins, rods, plates and nails). However, in cases where multiple fragments of bone have resulted from multiple breaks, there is currently no convenient way to stabilise the small fragments of the fractured bone and prevent gaps between the bone fragments. An alternative approach to overcome some of the challenges relating to the use of metal hardware in fracture repair is the use of adhesive materials. Such materials are capable of stabilising the fractured bone, creating a bond between the metal implant and bone, or bone and bone [9]. However, potential drawbacks relating to the use of current adhesive materials include inflammatory responses, stress shielding and mechanical failure that can lead to premature implant failure [10,11]. Recently, to overcome these drawbacks, research relating to bone fracture healing and fixation has focused on the development of bioinspired adhesives based on the behaviour of terrestrial organisms and marine animals. This review article systematically describes complex bone fractures and the limitations of the currently used surgical method for bone fracture treatment. Additionally, this review presents a synopsis of existing and in development adhesives that meet clinical requirements, glue bone fragments easily and rapidly, and provide bone stabilisation without the need for removal after bone healing.

## 2. Complex Bone Fractures

**Figure 2:**
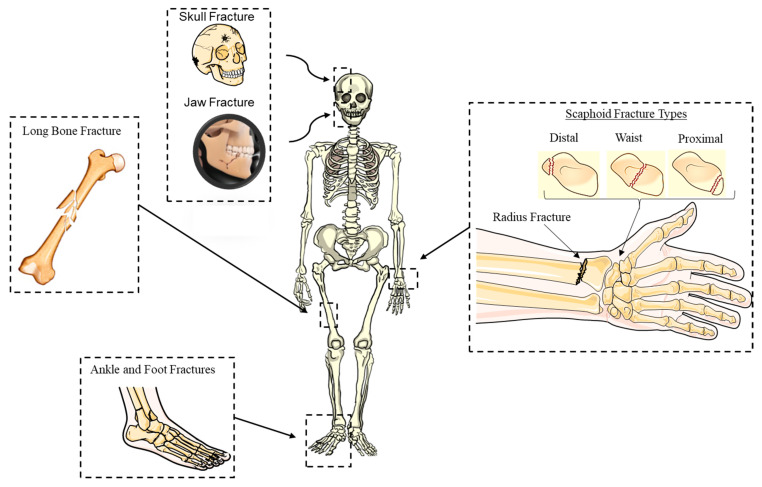
Complex fractures occur most frequently in the long bones, carpal, facial and ankle–foot bones. The wrist, facial and ankle–foot bones contain several small bones close to each other, leading to complex fractures with several bone fragments after a fracture.

Complex bone fractures generally consist of multiple fragments and usually require complicated surgical intervention (Figure 2). These fractures, therefore, present significant challenges for orthopaedic surgeons [12] and often lead to poor clinical outcomes. Complex fractures can vary significantly from one patient to another and may be further complicated due to joint dislocation and loss of bone fragments, leading to a painful and difficult recovery process for the patient [13]. The most common types of challenging bone fractures are distal radius fractures [3], facial bone fractures [14] and foot/ankle bone fractures [15]. Currently, 20% of distal radius fractures [16] and 71% of facial fractures require surgical intervention, with almost 20% of facial fracture requiring secondary surgical procedures [17]. The incidence of fractures that require surgical intervention is reportedly increasing among the younger patient population, with 45% of fractures in the age group under 25 years requiring surgical intervention and 37.5% of fractures in the age group 25–30 years [17].

**Figure 3:**
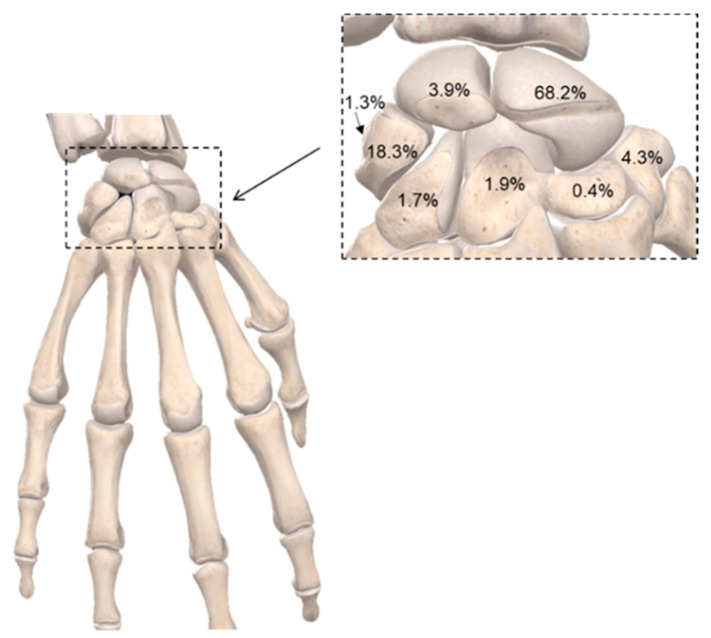
Percentage of fracture incidences per carpal bone.

An analytical distribution of wrist fractures, as well as the eight carpal bones of different shapes and sizes, can be seen in Figure 3. Scaphoid fractures are the most common carpal bone fractures (70% of all carpal bone fractures) [18] that cause long-term pain and frequently require surgery. The remaining 30% of carpal bone fractures are divided across the other six bones of the wrist and can cause significant disability. Trapezium fractures can occur within the body of the trapezium or at the ridge and usually result from a direct blow or an avulsion injury [19]. 

Facial bone fractures occur frequently, with an increased number of fractures being reported annually [20,21]. Facial fractures are categorised as: (1) isolated with lower energy trauma or (2) complex. In terms of the isolated fractures [22], the most common type is the fracture of the nasal bone, accounting for 40% of the cases, followed by mandible fracture at 30%. The fracture of the inferior region is the most common type of complex injury, with 14%—the highest frequency—being a tripod fracture (zygomaticomaxillary complex fracture, also known as a quadripod fracture, quadramalar fracture) [22]. 

It is estimated there are nine million incidents of long bone fractures worldwide per annum [23] caused by medical conditions (e.g., osteoporosis). According to Fisher et al. [24], 20% of incidents result in one or more complications such as deep infections (i.e., pain, erythema and pus discharge), fixation or implant failures (i.e., loosening of the screws and re-fracture following mobilisation), delayed union/non-union due to deep infection or failure of implant/fixation and re-fracture through the site of original injury or the screw hole. Treatment of long bone fractures at more than one anatomical site presents many clinical challenges and requirements due to the weakness of the osseous tissue [25], which ultimately leads to poor clinical outcomes [26]. Another fracture that appears complex and challenging to manage and treat due to the complexity of the bone anatomical site is the proximal humeral fracture [27,28]. Conventional surgical treatment for fracture of the proximal humeral bone normally leads to reduction in range of motion, poor restoration of anatomical congruity, pain and the likelihood of infection [27]. A common problem encountered by athletes of all levels and ages is fractures of the foot and ankle. The navicular, talus, medial malleolus, proximal fifth metatarsal and sesamoid bone fractures, due to the rate of non-union, are high-risk and require surgical fixation, with long periods of no load-bearing activity [28]. As complex fractures are very painful and difficult to recover from, the treatment plan must be carefully designed to achieve the best clinical outcomes. 

## 3. Current Surgical Approaches for Fracture Repair

Metallic plates and wires have been used to provide compression and stabilisation between the fractured bone fragments in internal fixation procedures for +100 years. Despite the widespread use of metal hardware, they have associated limitations and frequently result in poor healing, such as mal-unions [29]. In particular, the loosening of bone plates, screws and pins often occurs over time post-surgery and, as a result, the removal of such devices is often recommended, which leads to cortical bone loss [30]. 

The objective of early fracture management is to control bleeding, prevent ischemic injury (i.e., bone death) and remove sources of infection such as foreign bodies and dead tissues [31]. Fracture management includes reduction of the fracture followed by maintenance of the fraction reduction using immobilisation techniques. Currently used immobilisation techniques range from the use of a cast or wrap (i.e., non-operative therapy) for simple fractures to the use of metal hardware (i.e., operative therapy). Surgical treatment approaches are aimed at establishing stability to the broken bones above and below the fracture site with internal or external support. Another purpose of surgical intervention is to supply the fracture site and surrounding soft tissue with blood and to remove the dead bone and any poorly vascularised or scarred tissue from the fracture site to encourage healing. Sometimes, healthy soft tissue along with its underlying blood vessels may be removed from another part of the body and transplanted at the fracture site to promote healing. Furthermore, bone grafts can be used to stimulate the healing response by providing bone-forming cells and supportive cells to stimulate bone healing (stem cell therapy). More complicated fractures require surgical intervention, such as open reduction and internal fixation (ORIF) or external fixation. 

### 3.1. Open Reduction and Internal Fixation (ORIF)

**Figure 4:**
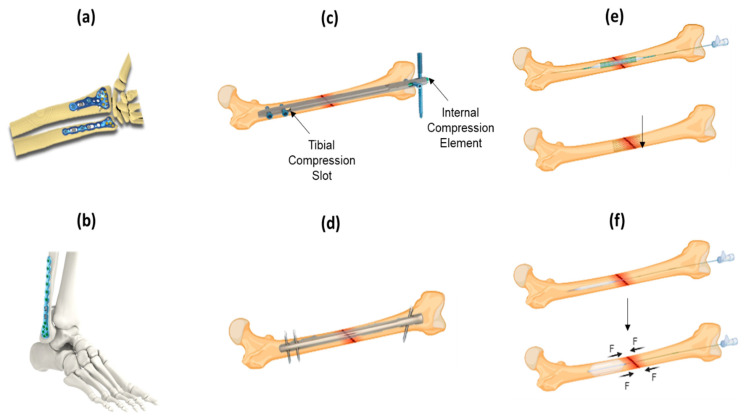
Internal fixation devices such as (**a**) dynamic compression plates for ulna and radius and (**b**) ankle bone fractures, including screws for the bone stabilisation, (**c**) intramedullary compression nail, (**d**) interlocking nail, (**e**) metallic stent to the fracture site “bridge” and (**f**) balloon application to the fracture site.

ORIF is a surgical procedure where the fracture site is adequately exposed, and reduction of the fracture is conducted. Several devices have been used for the internal fixation of bone fractures, including plates, interlocking nail devices, intramedullary compression nail devices, bridging devices and balloons [32]. There are a number of different types of plates, with the most common being dynamic compression plates. Dynamic compression plates (Figure 4a,b) are designed to exert dynamic pressure between the bone fragments, which is achieved either by attaching a tension device to a plate or by using a special plate. For the placement of the tension device, a longer surgical incision is required, and there is a possibility of re-fracture after the plate is removed. The benefits of dynamic compression plates include low incidence of mal-union and stable internal fixation, allowing immediate movement. However, the use of dynamic compression plates for fracture repair has several disadvantages, such as delayed union, existence of microscopic fracture gaps and cortical bone loss after plate removal [30]. For instance, Mardam-Bey et al. reported outcomes for tibial eminence fracture repair using screw fixation on dynamic compression plates, reporting that 20% of patients show anterior screw relaxation following treatment and 10% of patients experience rotational instability and loss of motion [33]. 

Intramedullary compression nails [34] (Figure 4c) and interlocking nails [35] (Figure 4d) are also widely used in bone fracture repair. The intramedullary compression and interlocking nail are inserted into the medullary cavity of a bone to rejoin and reinforce the broken bone parts and permit the functional rehabilitation of the limb within a few days. These nails usually do not demonstrate sufficient mechanical strength to enable full load-bearing capability, therefore, functional use of the limb is not possible until the healing process is complete. Consequently, immobilisation of the limb for long periods is required, which can impact the patient’s quality-of-life and ability to work during that time and also poses risks of muscle atrophy and other ailments. The interlocking nail method is frequently used for the treatment of complex and unstable fractures of the femoral shaft. This is a technically challenging procedure due to the requirement for accurate placement of locking and stabilisation screws that secure the compression nail in place.

A bridging device is an expandable fracture fixating device used for internal fixation by implanting the device within the medullary cavity (marrow conduit) of the bone and positioning it across the fracture (Figure 4e) [36]. These expandable and hollow structures are able to “bridge” the bone fracture site, fixate the site upon expansion and allow the maintenance of the majority of the bone marrow volume. Their use has been shown to enhance bone health, healing and the ability of the body to generate red blood cells [37]. This device can be implanted for the temporary stabilisation and fixation of bone fractures, but after a period, surgical removal is required. A similar method developed by Berger et al. involved the use of a balloon catheter fixation device [38]. In this approach, a balloon catheter was placed either proximal or distal to the fracture site, adding compressive force to enable reduction and stabilisation of the fracture (Figure 4f). The main objective of these devices is, firstly, to stabilise the fracture site and, secondly, to increase the rate of healing. The elastic property of the catheter that is tightened against the rigid immobile force of the anchoring balloon allows the fractured segments of the bone to align and come in intimate contact. 

While these expandable fixation devices are considered to be minimally invasive, they are limited to long bones only, due to their length. Additionally, complications may occur, such as persistent infection (e.g., chronic osteomyelitis) of bone or bone marrow, since it requires delivery and penetration into the medullary cavity. Treatment of such infections requires hospitalisation and treatment with antibiotics or surgical drainage and curettage [32]. Post-surgical infections are one of the major complications associated with the application of all internal fixation devices. Frequently, these infections result in bone or tissue necrosis and, in severe cases, can result in the death of patient—therefore, additional surgical intervention and therapy are required. Although most bone fractures heal without complications, in some cases, successful healing is not achieved, resulting in delayed unions or non-unions, necessitating a bone graft.

### 3.2. External Fixation

**Figure 5:**
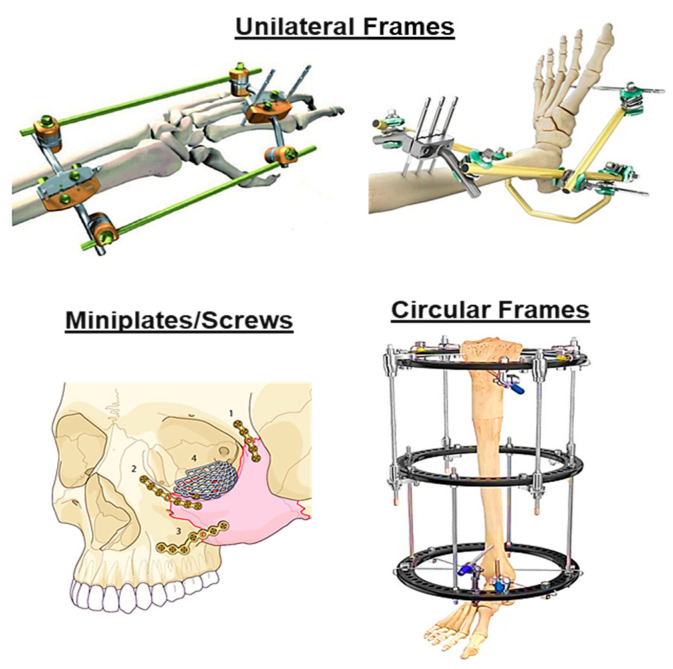
External fixation devices such as unilateral frames [41] placed on one side of the ankle or distal bone and miniplates or screws including pins for molar bones and circular frames [42] placed around the long bone, allowing functionality and bone stability during the bone healing process.

External fixation is a procedure in which the fracture stabilisation is achieved at a distance from the site of fracture. It helps to maintain bone length and alignment without casting. Devices used for external fixation are made of metal or carbon fibre and, as with skeleton traction methods, these devices have pins placed into the bone directly through the skin [39]. External fixation has evolved from being used primarily as a last resort fixation method to becoming a mainstream technique used to treat bone and soft tissue pathologies. Percutaneous techniques are used for the treatment of tibia periarticular [40] and femoral shaft [35] complex fractures, leading to the enhancement of biologic fracture healing and a decrease in the complications observed with other open reduction techniques. Development of unilateral frames, circular frames and miniplates or screws [39] have been reported (Figure 5). Unilateral and circular frames are positioned on one side of or around the limb with the use of pins, allowing the limb to remain functional, avoiding the complications associated with immobilisation and providing bone stability. However, these techniques are characterised by a high risk of wound and pin tract infection and incisional morbidity as well as damage to surrounding tissue, nerves, skin, and blood vessels or nearby organs [32]. Furthermore, these devices require substantial attention and care to prevent inflammation. 

## 4. Bioadhesives 

To date, a range of synthetic, naturally-derived and biomimetic-based adhesives have been developed for use in a range of clinical applications, including bone repair. They include calcium phosphate cements [43], cyanoacrylates [44], polyester cements [45], poly(methyl methacrylate) (PMMA) bone cements [46] and fibrin [47].

### 4.1. Synthetic Bioadhesives

#### 4.1.1. Cyanoacrylates

**Figure 6:**
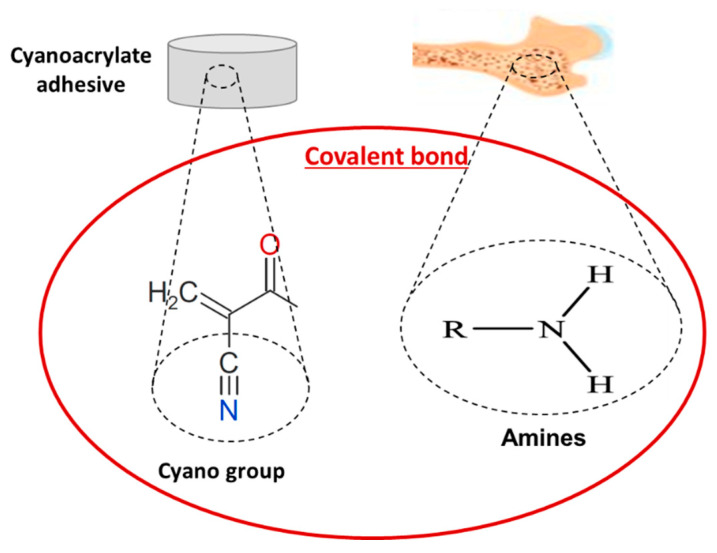
Covalent bond between cyano groups of the adhesive system (cyanoacrylate-based) with amines present in bone collagen matrix.

Cyanoacrylates were one of the first synthetic adhesives used as bone adhesives, demonstrating a high potential for bone bonding, together with methacrylates. Cyanoacrylate adhesives are very promising due to their ability to polymerise under wet conditions (e.g., existence of blood) and to achieve strong wet adhesion and, at the same time, via covalent bonds (Figure 6), they are able to adhere themselves with the amines on the surface of the tissue, achieving rapid curing at low cost [48]. However, the rapid polymerisation leads to an exothermal reaction that has been shown to result in the formation of a hard and brittle film on the bone, leading to cell death and tissue damage [49]. The adhesive strength provided by cyanoacrylate-based adhesives is generally reported to be lower than the bonding and fixation strength achieved using screws [50]. However, a study by Kandalam et al. explored the use of a N-butyl cyanoacrylate for the replacement of screws and plates in pig cortical bone samples and reported a higher range of shear strength (1–2 MPa) compared to that achieved using a plate and screw system (0.49 MPa) [51]. 

Despite the enhanced mechanical properties and the ability for adhesion in wet environments, the clinical use of cyanoacrylate-based adhesives is limited due to the toxic nature of the degradation products, which result in a chronic inflammatory response, tissue necrosis and dermatitis in vivo and cytotoxicity for cells in direct contact in vitro [52]. Lee et al. [53] compared the biocompatibility of prepolymerised allyl 2-CA (PACA)-based tissue adhesive with commercial available cyanoacrylate–based adhesive (e.g., *Dermabond*, Johnson & Johnson, New Brunswick, NJ, USA) and demonstrated that both adhesives were cytotoxic. However, a lower cytotoxicity and reduced tissue inflammation was observed using the PACA-based adhesive compared to the cyanoacrylate-based adhesive. In addition, despite achieving good fixation without displacement or detachment, high cytotoxicity was observed for both the unpolymerised and polymerised cyanoacrylate-based adhesives in vivo in a rabbit subcutaneous model by Pascual et al. [54]. The high cytotoxicity obtained from cyanoacrylate-based adhesives is due to the short alkyl chain length. Even though both n-butyl-2-cyanoacrylate (NBCA) and octyl-2-cyanoacrylate (OCA) are considered harmless and non-carcinogenic, there is no FDA (Food and Drug Administration) approved bone adhesive based on cyanoacrylates. In order to enhance the clinical and mechanical properties of synthetic polymers, various types of biodegradable ceramics and glasses have been added. For instance, bioactive glasses, due to their excellent osteoconductivity [55], have been encapsulated and combined with octyl cyanoacrylate, aiming to increase the migration of bone-derived mesenchymal stromal cells into the adhesive layer and promote their differentiation into osteocytes [56,57]. While instant bonding with high mechanical properties and high efficiency of bone regeneration was achieved, the toxicity of the octyl cyanoacrylate limited further improvement. Furthermore, a hydroxyethyl methacrylate (HEMA) adhesive reinforced with bioactive glass nano particles was developed, demonstrating double tensile strength and significantly enhanced biomineralization and biodegradation compared to the pure HEMA adhesives [58]. Excellent mechanical properties and osteoconductivity can also be achieved with the addition of different calcium phosphates, such as nano-hydroxyapatite [59]. This study combined a biodegradable polymer and an acrylic polymer augmented with bioactive nano-hydroxyapatite; histological results provided high biocompatibility and osteointegration with improved bioactivity [58].

#### 4.1.2. Polyurethanes

**Figure 7:**
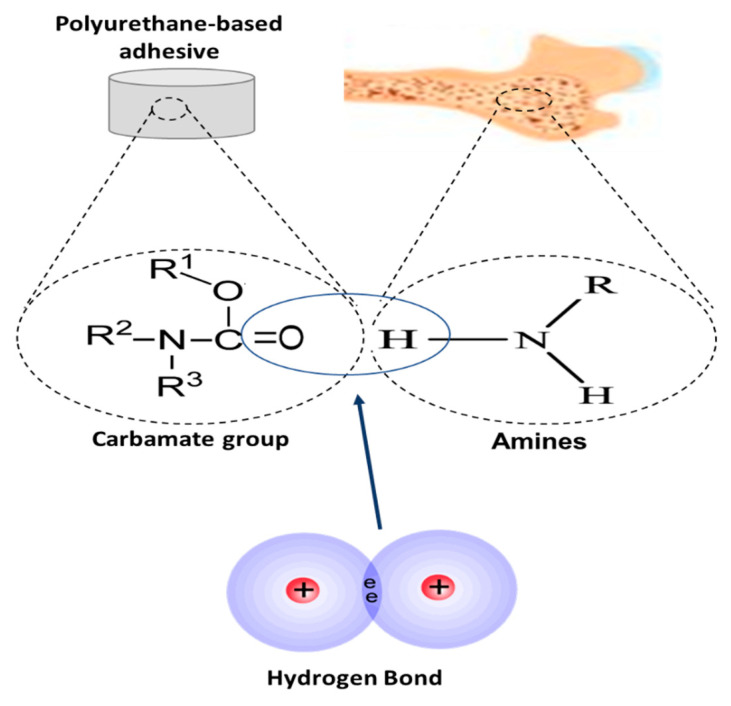
Chemical and/or physical bonding of polyurethane-based adhesives with bone. Hydrogen bonding occurs between the carbamate group of adhesive system and the amines present in bone collagen matrix.

Polyurethanes are produced by combining polyisocyanates and polyols in the presence of a catalyst or ultra-violet light. Polyurethane-based adhesives have shown promise for orthopaedic applications as they are biocompatible and demonstrate a high adhesion strength, which is achieved through chemical and/or physical bonding between bone and the adhesive (Figure 7). For example, a polyurethane-based adhesive led to a successful adhesion of bone with a high tensile and adhesion strength on unprimed and primed bone, however, it demonstrated limited biodegradability [60,61]. Changing important factors such as molecular composition, degree of crosslinking, active chemical groups and molecular stiffness can lead to a significant change in the bonding within these polymers and, as a result, can improve biodegradation. To date, a minimal degree of biodegradability has been achieved, which has largely been reported to occur via either a hydrolysis or enzymatic process [62]. The successful closure of bone fractures using a polyurethane-based adhesive without any reaction has been reported in vivo—however, mechanical and functional performance under in vivo conditions was not investigated. Despite advances, currently, the main drawbacks of polyurethane-based adhesives (e.g., premature failure, interfacial bond failure between bone and adhesive, wound infection and tissue necrosis) outweigh the benefits (e.g., high adhesive and/or cohesive strength, osteogenic, non-toxic, high workability and the ability to be delivered by minimally invasive means). As a result, their use in biomedical applications was discontinued in 1990 when a formulation of a novel non-elastomeric polyurethane-based adhesive with calcium and phosphate was developed [63]. Furthermore, in 2012, an FDA approved castor oil-derived polyurethane-based cement, *Kryptonite^TM^* (Doctors Research Group Inc., Southbury, CT, USA), was recalled by the FDA because it failed to meet the necessary clinical standards in terms of product safety, as well as its exceptionally long hardening time [9,64].

#### 4.1.3. Polyesters

In bone tissue engineering applications, the resorbable aliphatic polyester poly(L-lactide) (PLLA) has been used as a scaffold in bone regeneration [65]. Copolymers of PLLA with superior mechanical properties have been developed as bone tissue engineered scaffolds, but the influence of copolymerisation, the osteogenic potential is unclear. For instance, biodegradable polymers that can be shaped in situ and adhere to living tissues were developed from the copolymerisation of D,L-lactide polymerisation or D,L-lactide-epsilon-caprolactone (50:50). These polyester copolymers demonstrated faster degradation under wet conditions compared to polyurethane copolymers [66]. In spite of the improved degradation properties compared to standard polyether copolymers, inflammation at the application site remains a limitation. Agarwal et al. [67] reported high adhesion strength for polyester-based adhesives. These adhesives demonstrated low yield strength and significant cytotoxicity during in vitro studies. Therefore, despite the enhanced functional properties of these adhesives, the limitations preclude use as an adhesive for bone tissue engineering applications. 

These types of adhesives continue to attract much attention, with recent studies focusing on the investigation and development of polyester-based adhesives leading to enhanced combined properties. Polyethylene glycol (PEG)-based adhesives comprised of PEG ester and glutaryl-succinimidyl ester have been tested for repair of cranial and spinal injuries. The PEG-based adhesive offered high bonding strength due to covalent bonding (i.e., between thiol group and carbonyl group of succinimidyl ester), as well as normal wound healing rates with no post-operative complications. As a result, PEG-based adhesives such as *DuraSeal™* (Covidien, Mansfield, MA, USA), which is composed of tetra-PEG-succinimidyl ester and trilysine amine, have been FDA approved and used for cranial surgery [68]. Since the synthesis of the first poly(glycerol sebacate) (PGS) as a tough biodegradable polyester in 2002, a number of modifications have been implemented to enable its clinical application [69]. Pure PGS modified and/or combined with other materials has achieved novel properties [70]. For example, with the addition of a thermoplastic polymer, poly(ε-caprolactone) (PCL), the PCL-modified PGS demonstrated good biocompatibility and cytocompatibility, higher mechanical properties, degradation rate and hydrophilicity [71], while the addition of PEGylated-CH nanoparticles to the PCL-modified PGS resulted in improved antibacterial properties, effective drug release and accelerated wound healing [72,73]. Moreover, good biocompatibility, decreased water contact angle, improved surface hydrophilicity and enhanced cell adhesion was achieved by incorporating poly (vinyl alcohol (PVA)) to PGS, resulting in a promising biodegradable PVA–PGS bioadhesive [74]. In addition to PVA–PGS, similar improved performance was achieved by blending PGS with different types of nanoparticles [75] such as PGS urethane (PGSU)/renewable cellulose nanocrystals (CNCs) [76] and hybrid elastomers PGS–silica glass. Specifically, PGS–silica glass modified adhesive demonstrated controlled production of matrix mineralisation with increased alkaline phosphatase (ALP) activity and osteoinductive capability, tunable elastic properties and biodegradation and enhanced osteoblast proliferation [77,78]. The incorporation of nanoparticles in the PGS offers a new choice for bone tissue repair and regeneration. For instance, the blending of PGS with β-TCP nanoparticles for guided bone regeneration resulted in a bioadhesive with improved mechanical properties and a controlled degradation rate [79]. PEGS/β-TCP promoted cell attachment/viability and superior bone tissue regeneration. Facilitation of the osteogenic differentiation was also observed due to the enhanced mineralisation and the ALP activity resulting from the presence of β-TCP.

#### 4.1.4. Poly-methyl Methacrylates (PMMA)

**Figure 8:**
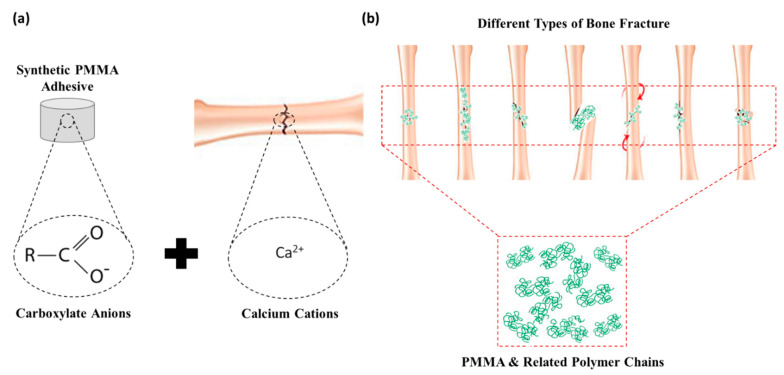
Mechanisms of action of PMMA-based adhesive materials. (**a**) Chemical and/or physical bonding through ionic interaction between carboxylate anions of adhesive system with Ca^2+^ present on the surface of bone and (**b**) mechanical interlocking through infiltration of the polymer chains into surface irregularities.

PMMA-based adhesives are the most commonly used adhesives in dentistry (since the 1930s) and orthopaedics (since 1958) for total joint replacement applications [80]. PMMA-based adhesives are used to support the prosthetic implant within the bone cavity, where they act as a grouting agent between the bone and implant, in addition to providing fixation [81,82]. Synthetic PMMA adhesives can create chemical and/or physical bonding through ionic interactions (Figure 8a), while PMMA-based adhesives can create a mechanical interlock between bones through the pressurised infiltration of the polymer into surface irregularities (Figure 8b). Even though PMMA-based adhesives are widely used, they exhibit low adhesive strength due to hydrophobic properties. Another drawback of these adhesives is that, in the absence of bone pretreatment or polymer chemical modification, the exothermal reaction that occurs during the polymerisation reaction can lead to considerable thermal necrosis of bone tissue [49]. The potential for carcinogenesis has not been associated with PMMA-based adhesives, although mutagenesis has been reported in bacteria [83]. Many attempts to overcome these challenges have been reported, such as the chemical modification of the PMMA combined with the enrichment of the cement with hydroxyapatite particles to enhance the functional properties [84]. The hydroxyapatite-modified PMMA cement showed higher adhesion than unmodified PMMA bone cement, being used as adhesives in dentistry, replacing the conventional PMMA adhesives. Despite clinical use, the lack of biodegradability of PMMA-based adhesives remains a significant limitation. 

Approaches to overcome these challenges have involved the synthesis of different copolymers with combination properties [85]. Initial attempts focused on the combination of methyl methacrylate reactivity with the biocompatibility and biodegradability of polylactides, since the mechanism of degradation is well established. The adhesive qualities of PMMA to bone have been improved through the use of liquid acrylic resin, phosphoric acid etching or tributyl borane [86]. Despite the synthesis of copolymers with PMMA, different polymerisation techniques have also been used to achieve favourable biocompatibility, biodegradability and improved adhesion [87]. These PMMA–based adhesives demonstrated acceptable biocompatibility and adhesion, while the degradation did not interfere with physiological fracture healing. While good short-term results have been reported with respect to the use of these adhesives in mandibular fractures, spine fractures and isolated long bone fractures, issues relating to late displacement and non-union have prevented clinical use as an adhesive for the treatment of bone fractures [88,89]. 

The different application sites as well as properties and drawbacks of the synthetic-based bone-adhesive materials described in this section are summarised in Table 1.

### 4.2. Naturally-Derived Bioadhesives

**Figure 9:**
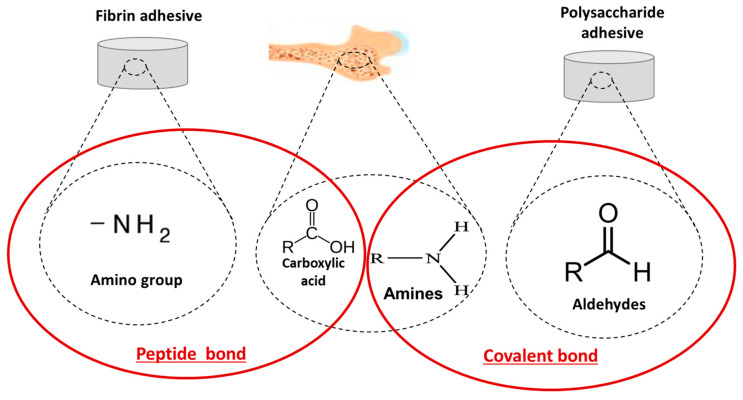
Covalent bond between amino groups of fibrin/fibronectin and/or aldehydes of polysaccharide-based adhesive system, with carboxylic acid groups and amines present in bone collagen matrix.

The first reported biological bone adhesive, which combined fibrous protein and collagen, was developed in 1931 [90]. The largest group of biologically derived adhesives and sealants is fibrin sealants. Other biological polymer-based adhesives include gelatin–resorcin–aldehyde adhesives, protein–aldehyde adhesives, collagen-based adhesives and polysaccharide-based adhesives. Naturally-derived bioadhesives create bonds with the bone through chemical and/or physical bonding due to amines and carboxylic acid groups present in the bone collagen matrix, respectively (Figure 9). In particular, a peptide bond (chemical bond) is formed when the carboxyl group of one molecule reacts with the amino group of the other molecule, releasing a molecule of water for fibrin adhesives while a covalent bond results in the creation of amines and aldehydes in polysaccharide-based adhesives.

#### 4.2.1. Fibrin 

Fibrin is a fibrous non-globular protein that plays a role in the promotion of blood clotting. Most fibrin-based adhesive systems are formed by combining a fibrinogen source and factor XIII as a stabiliser [91]. Thrombin, calcium and an anti-fibrinolytic agent can also be incorporated to prevent rapid fibrinolysis. The combination of thrombin and fibrinogen in the presence of calcium ions enables activation and allows for clot formation. Adhesion is achieved through the formation of a covalent bond between the amino groups of fibrin/fibronectin within the adhesive system and carboxylic acid groups present in bone collagen matrix [48]. In terms of bone fixation, fibrin-based adhesives can accelerate the fixation of implants to bone, improve bone graft filling, fracture fixation and spinal fusion [92]. 

Despite good biocompatibility, biodegradability and clot formation, these types of adhesives demonstrated a lack of osteogenic potential and a relatively low bond strength compared to synthetic adhesives of 0.005–0.17 MPa [81], which can be attributed to the low cohesive strength within the fibrin itself. The use of fibrin sealants is limited to fractures where there are no mechanical forces applied to the fragments within the application site, since they are unable to withstand significant tensile forces [93,94]. The fibrin-based adhesives are divided in two types: (1) allogenic and (2) autologous fibrin sealants. Autologous fibrin sealants have major implications for use in orthopaedic surgery [90]. However, fibrin-based adhesives have many advantages over synthetic-based adhesive systems such as cyanoacrylates, in view of their excellent biocompatibility, biodegradability and cost effectiveness. Therefore, these materials have been extensively used in orthopaedic surgery. For the optimal use of fibrin-based sealant systems, specific requirements need to be met during clinical application. For example, the wound surfaces should be dry, and the sealant should be applied as a thin film at 37 °C. After clotting has occurred, further mechanical stresses should be avoided for approximately 5 min. The first FDA approved fibrin glue was *Tisseel^TM^* (Baxter Inc., Deerfield, IL, USA) in 1998. Since then, the FDA has approved several fibrin-based adhesive products, including *TachoSil^®^* (Corza Health, Inc., Osaka, JAPAN), *Vivostat^®^* (Vivostat, A/S, Alleroed, FRANCE), *Evicel^®^* (Omrix Biopharmaceuticals, Machelen, BELGIUM), *Cryoseal ^®^* (Thermogenesis, Rancho Cordova, CA, USA) and *Vitagel^®^* (Orthovita, Malvern, PA, USA) [95]. However, all these fibrin-based products are indicated for use as an adjunct to standard surgical methods to control bleeding.

#### 4.2.2. Gelatine–Resorcinol–Aldehydes

Gelatine–resorcinol–formaldehyde adhesives were first developed as haemostatic agents (1966) and as a tissue adhesive (1979) [96,97]. While gelatine–resorcinol–formaldehyde adhesives have not been clinically tested as bone adhesives, in vitro testing shows that they are stronger than fibrin sealant with water resistance but less strong than many available synthetic-based adhesives [98]. In vitro, the bond strength to bone achieved using these adhesives has been reported to be approximately 0.2 MPa [99]. Studies also report that these materials demonstrate lesser tissue irritation than cyanoacrylates and higher bond strength, tensile strength and tissue compatibility when compared to methylcyanoacrylates [99,100]. Furthermore, recent studies have focused on the modification and enhancement of gelatin-based adhesives to achieve lower swelling, improved degradability and low cytotoxicity. In particular, Liu et al. developed a gelatin-based adhesive crosslinking catechol-modified gelatin (Gel-Ca) and phenol-modified gelatin (Gel-Ph) for wound healing applications. The gelatin-based adhesive demonstrated improved mechanical and rheological properties when compared to other recently reported ion-crosslinked catechol-modified gelatin adhesives [101].

#### 4.2.3. Polysaccharides

Polysaccharides, such as chitin, chitosan, chondroitin, dextran or starch, are an important class of soft/hard tissue adhesive and haemostatic material. They are relatively easy to prepare and apply and can generate biocompatible and biodegradable properties. Chitosan-based adhesives are known for their haemostatic properties and are commercially available for bone [102], cartilage [103] and soft tissue [104] repair. Hoffmann et al. [105] combined chitosan with starch to develop a bioadhesive system that has potential for use as an emergency haemostasis agent as well as for skin wound closure. The bonding mechanism is achieved through the formation of covalent bonds between aldehyde groups with amino groups present in surrounding tissues or exposed in the fractured bone, enabling a strong bonding to tissue [61]. Further in vitro studies demonstrated that these adhesives are biocompatible, with an adhesive strength between 40 ± 1.09 MPa and 45 ± 1.02 MPa [105]. Degradation studies of saccharide-based adhesives indicated a mass loss of 10–15% within the first 24 h [106]. Further optimisation of these materials is required to reduce the degradation rate for bone tissue engineering applications. Although there are a series of polysaccharide-based materials on the market that can be used in both wet and dry environments, some important issues relating to biosafety, haemostatic effect and high cost still greatly limit their widespread use in biomedical applications [107]. 

### 4.3. Biomimetic-Based Adhesives

**Figure 10:**
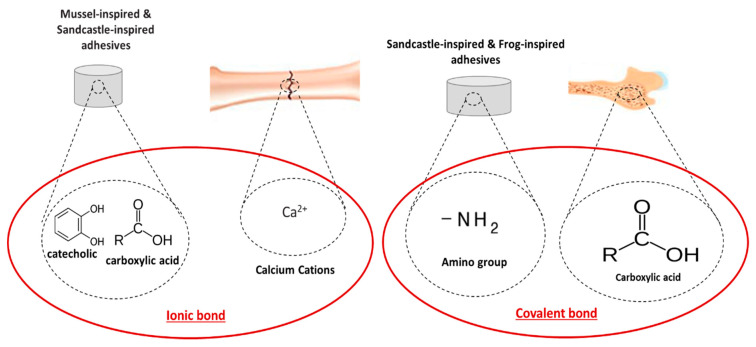
Ionic bond between catecholic hydroxyl and carboxylic acid groups of adhesive systems with Ca^2+^ present on the surface of bone as a mechanism of adhesion of mussel- and sandcastle-inspired adhesives, and covalent bond between carboxylic acid of adhesive system with amines present in bone collagen matrix for frog- and sandcastle-inspired adhesives.

Some terrestrial organisms as well as marine plants and animals use combinations of proteins and polysaccharides for the formulation of bioadhesives to meet specific requirements to function in the natural environment (e.g., settlement, hunting and defence) [61]. In many cases, these bioadhesives demonstrate higher mechanical properties compared to the currently developed synthetic or natural polymer-based adhesives and adhesion within a wet environment. Specifically, these types of adhesives are able to create ionic and/or covalent bonds with the bone surface or bone collagen (Figure 10). The ability to cure at physiological temperatures and to achieve a high bonding strength to biological materials including bone materials has prompted research into its use as a bioadhesive for bone tissue engineering applications. To date, a number of bioadhesives that mimic these animals and plants have been investigated and/or developed, but the bioadhesives produced have not yet been translated for clinical use for bone tissue engineering applications. The different types of biomimetic adhesives discussed and their properties are summarised in Table 2.

#### 4.3.1. Terrestrial Organisms-Inspired Adhesives

There are a number of terrestrial organisms that are capable of forming bioadhesives, including the Australian frog (e.g., *Notaden bennetti*) and Caddisfly (e.g., *Trichoptera*). The *Notaden bennetti* can form a protein-based elastic hydrogel-based adhesive that is able to function in moist environments and bind to biological tissues as well as other surfaces [48]. The bonding is achieved by covalent bonding with amines present in the bone collagen matrix. These frog-derived bioadhesives performed significantly better than fibrin glues in cartilage repair models, providing biocompatibility and resorbability, although they did not outperform cyanoacrylates in terms of adhesion strength [108]. Overall, the unique properties of these biomimetic copolymers suggest that they could have great potential for application as bioadhesives for bone tissue engineering applications. However, the research related to this bioadhesive is still at a primary stage, and further investigation is required to evaluate this material as a bioadhesive for bone fragments’ stabilisation and repair [81]. Stewart et al. described a bioadhesive that mimics caddisfly silk, combining phosphate-functionalised and amino acid-based poly(ester urea) copolymers for the enhancement of the mechanical properties [109]. These bioadhesives demonstrated higher levels of adhesion to bovine bone when crosslinked with Ca^2+^ ions. 

#### 4.3.2. Marine Animals-Inspired Adhesives

Marine animals, such as the blue mussel (e.g., *Mytilus edulis*), barnacle (e.g., *Balanus hameri*) and the sandcastle worm (e.g., *Phragmatopoma calfornica*), also produce adhesive proteins. *Mytilus edulis* have the ability to strongly attach themselves to both inorganic and organic host surfaces at various levels of salinity and humidity at ambient temperature [110]. The functionality of these mussel-derived bioadhesives is based on an extremely complex interaction between different proteins. These bioadhesive usually consists of four main components: (1) acid mucopolysaccharides acting as a primer, (2) polyphenolic proteins as adhesive proteins rich in both 3,4-dihydroxyphenylalnine (L-DOPA) and lysine, (3) fibrous proteins between mussel and the substrate as an attachment thread and (4) polyphenoloxidase to promote intermolecular cross-linking [111]. In the context of bone repair, adhesion is achieved through ionic bonding between catecholic hydroxyl and carboxylic acid groups of the adhesive system with Ca^2+^ present on the surface of bone. The complex interactions between the proteins’ complex within mussel-derived bioadhesives causes technical difficulties relating to protein extraction, resulting in high production costs that hamper clinical application. Many studies have been conducted to evaluate the properties of mussel-derived bioadhesives [112]. Initial efforts to mimic these materials have focused on the development of synthetic polymers and cell attachment proteins that mimic the components that provide mussels with strong adhesion. Mussel-derived bioadhesives assessed for bone tissue engineering applications have demonstrated good biodegradability, non-immunogenicity and a greater adhesion on various substrates (e.g., metal, glass, plastic and biological substances) [113,114] compared to polymer-based adhesives. The mechanical properties of mussel-derived bioadhesives include an adhesion strength of 10 MPa, low Young’s modulus of 0.9 GPa and residual resilience of 53% following mechanical assessment under fatigue loading. Initially, pre-modified intestinal bacteria combined with an enzyme capable of inserting in the amino acid named DOPA (a key component in the mussel proteins) was developed, using photochemical crosslinking [115]. Apart from photochemical crosslinking, mussel-derived bioadhesives can be successfully crosslinked using oxidation agents (e.g., iron). Iron-induced networks showed strong adhesion, biodegradability, low cytotoxicity and a low exothermic reaction suitable for the bonding of sternal bones [116]. Furthermore, positive results were exhibited in terms of the suitability of these mussel-derived bioadhesives for bonding titanium prosthetic implants to bone. Other bioinspired approaches include the use of allyl, methacrylamide and thiol groups for bone priming, using a layer-by-layer coating technique leading to improved shear strength (0.3 MPa) and cellular response [117]. Drawing inspiration from the mussel-derived bioadhesives, further research is on-going to investigate the incorporation of DOPA into a range of different synthetic polymers to synthesize new copolymers with adhesive properties. Researchers have demonstrated that the bonding strength increased as a function of DOPA content, copolymer solution concentration, copolymer molecular weight and curing temperatures or by incorporating a crosslinker (e.g., tyrosinase, hydrogen peroxide, or basic aqueous solution) [115,118]. While the capability of these bioadhesive to bond various materials has been demonstrated, their suitability as bioadhesives for bone tissue engineering application is still under investigation.

Nishida et al. synthesised a synthetic-based bioadhesive that mimics the *Balanus hameri* barnacle, which demonstrated a tensile shear strength of ~2 MPa when bonding iron substrates [119]. Different amino acid compositions were used for the bioadhesive formulation, however, all the model peptides exhibited poor adhesion to bovine bone, and with the strongest bond strength achieved being ~ 363 kPa [120]. To improve the adhesion and tensile strength, a polyacrylamide-based copolymer with hydroxyl and hexyl groups for surface interaction and tetra-alanine groups for crosslinking has been developed to mimic the barnacle adhesive [121]. 

Another marine creature which has inspired the improvement of bioadhesive properties is the sandcastle worm (i.e., *Phragmatopoma calfornicaI)*, which produces an adhesive commonly known as ‘sandcastle glue’, comprised of polyphenolic proteins. The sandcastle worm produces an adhesive that can bind seashell fragments, grains and sand to each other. The maximum adhesion strength of this adhesive is achieved in less than 30 s in water, and it fully hardens within 1–2 h [122]. Cost-effective adhesion can be achieved using only small amounts of the secreted adhesive instead of typical amounts of 5 g to 10 g required for other adhesives. The glue includes phosphate and amine side groups, which are well-known bioadhesive groups that can be used for bone tissue engineering applications. The suitability of this bioadhesive for underwater adhesion makes this hybrid naturally-derived model an attractive potential bioadhesive for the stabilisation and repair of hard tissue (e.g., bone). A range of synthetic-based materials which mimic the adhesive function of the sandcastle glue has been developed. For instance, Ailei Li et al. developed a sandcastle glue-derived copolymer using bone block specimens from bovine femur cortical bone which exhibited an in vitro bone-bond strength of 0.1 MPa [123]. Another sandcastle worm-based bioadhesive was developed by combining O-phospho-L-serine, which is a phospho-related amino acid component of many proteins, with tetracalcium phosphate [124] or alpha-tricalcium phosphate [125]. This bioadhesive provided high levels of bone-to-bone bonding with a fast setting in a wet environment. Furthermore, the shear strength observed was 10-fold higher than PMMA-calcium phosphate-based bioadhesives and 40-fold higher than commercial cyanoacrylate-based bioadhesives, with an appropriate biodegradation rate that promoted osteointegration and supported effective bone ingrowth.

**Table 2:** Comparison of the different natural-based adhesives.

Biomimetic Adhesives				
	Description	Application	Advantages	Disadvantages
*Notaden bennetti* frog bioadhesives [81,108]	Protein-based elastic glue	Bone adhesion and fragments fixation (cartilage bone repair) Binding to biological tissues as well as other surfaces	Better biocompatibility and biodegradation than fibrin glues Function in moist environments	Lower adhesion strength than cyanoacrylates
Caddisfly silk bioadhesives [109,110,124]	Phosphate-functionalised and amino acid-based polyester copolymers	Bovine bone adhesion (orthopaedic) Scaffold materials for spinal cord injury Mesh grafts to treat hernias, ulcers and burns	Adhesion strength of 1.17 MPa Biodegradable in vitro and in vivo Higher interface compliance	Cohesive failure Low curing kinetics and adhesive properties on translationally relevant substrates
*Balanus hameri* barnacle bioadhesives [119,121,126]	Polyacrylamide-based copolymer with hydroxyl and hexyl groups	Repeatable and robust underwater adhesion to various substrates Material transfer, temporary fixation (orthopaedics) and material separation Bovine bone adhesion	Tensile shear strength of 2 MPa Enhanced toughness and cohesion strength Good elastic properties Rapid and reversible adhesion in water	Poor adhesion to bovine bone approx. 363 kPa Low mechanical strength
*Mytilus edulis* blue mussel bioadhesives [112,113,117,118]	Adhesives based on complex interaction between different proteins	Strong attachment to inorganic/organic surfaces at dry/wet environment Reliable crosslinking using oxidation agents, such as iron Suitable for joining titanium implants to a bone and/or bonding sternal bones	Non-immunogenicity and low cytotoxicity Greater adhesion on various substrates with adhesion strength of up to 10 MPa Good biodegradability Low exothermic reaction for the bonding of sternal bones	Difficulties relating to protein extraction resulting in high production costs, hampering the practical use Further research needed to determine the suitability of this adhesive as bone adhesive
*Calfornica* sandcastle worm bioadhesives [123,124,125,127]	Polyphenolic protein and phosphoserine-based adhesive	Strong attachment in a wet environment Reconstruction of craniofacial fractures Bonding of wet bone fragments Bond tissues to metallic and polymeric biomaterials	Maximum adhesion strength and hardness in <30 s Osteointegration, bone ingrowth and resorbability Small amount of adhesive needed to achieve the optimal properties Biodegradable and osteoconductive	Further in vitro and in vivo studies need to be conducted to verify the suitability to natural bone adhesion

## 5. Clinical Requirements of Bioadhesive for Bone Fracture Repair

**Figure 11:**
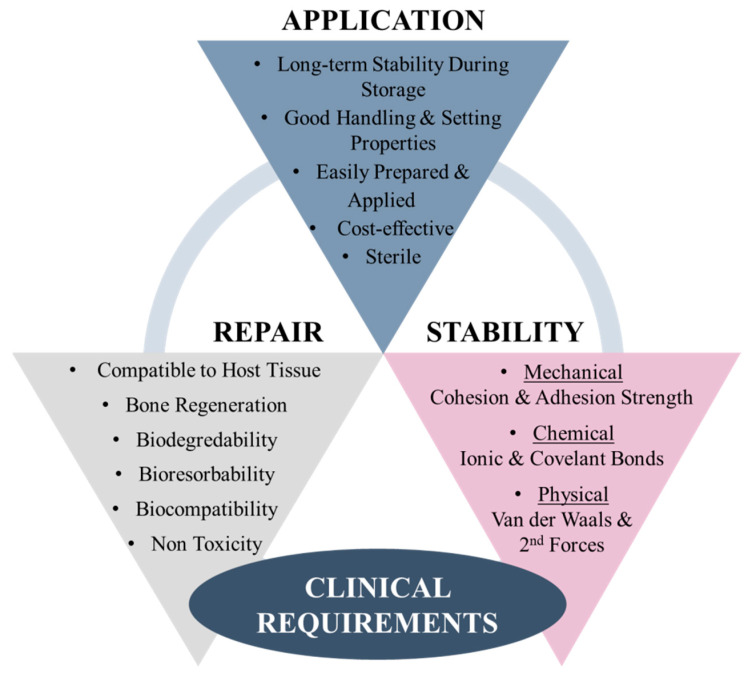
Clinical requirements for suitable application of adhesive for bone repair and stabilisation.

Bioadhesives present a promising approach for bone fracture stabilisation, repair and regeneration applications, with the potential to overcome the limitations of existing fracture repair techniques. In addition to the clinical imperative to develop adhesives that can replace the surgical requirement for metal hardware, there is also a high demand for the development of an adhesive that could be used in conjunction with traditional metal hardware to improve fracture stabilisation and potentially reduce the risk of micromotion and loosening of these devices over time. In order for a bone adhesive to be suitable for use in bone fracture stabilisation and repair applications, it must meet several clinical requirements (Figure 11) [48]. In particular, adhesives must provide early mechanical stability, combining optimal adhesive and cohesive properties. Appropriate adhesion to the bone under clinically relevant situations such as a moist environment, presence of bleeding and uneven surfaces, as well as stability under internal or external forces (e.g., tensile, compression or shear forces), must be achieved. Biocompatibility is also an important requirement in order to avoid cytotoxic responses and facilitate fracture healing through osteogenesis and, ultimately, bone regeneration. The adhesive also needs to be biodegradable and bioresorbable with non-toxic by-products such as gases (e.g., CO_2_), water and inorganic salts that can be processed naturally by the body without causing cytotoxic effects.

## 6. Bioadhesives for Bone Fracture Repair

A number of the synthetic, naturally-derived and biomimetic-based adhesives that have been previously discussed have been explored and adapted for use in bone repair applications, including fracture fixation, bone defect repair and prosthetic implant bonding to soft/hard tissue. These bioadhesives have the potential to overcome the disadvantages of conventional invasive surgical techniques and meet clinical requirements. Early investigations into the use of bioadhesives in bone repair applications involved the development of epoxy resin-based bioadhesives, such as phenol–formaldehyde resins. While these materials offered a high mechanical strength, they have been reported to lack biocompatibility [128]. Cyanoacrylate- (e.g., cyacrin) and polyurethane-based synthetic polymers have also been proposed as bone bioadhesives due to their high bonding strength and ability to achieve adhesion in a wet environment [129]. However, these cyanoacrylate- and polyurethane-based bioadhesives have demonstrated high tensile and adhesion properties—high infection rates, non-union (e.g., fracture displacement), low biodegradation and severe local reactions have been reported [54,128]. The poor outcomes from these initial materials resulted in research into alternative bioadhesives with more suitable functional properties and improved clinical outcomes.

One such study investigated the application of a non-elastomeric crosslinked polyurethane-based bioadhesive for the stabilisation and repair of bone fragments from the tibia [63]. This bioadhesive was developed via the reaction of a polyisocyanate and polyol in conjunction with a catalyst. The bioadhesive was improved by incorporating calcium and phosphate compounds. In vivo results demonstrated that stabilisation and bonding of the bone fragments as well as a de novo bone growth were achieved, with no evidence of inflammation/infection at the fracture site, as well as some biodegradation and good biocompatibility [63]. A similar polyurethane-based bioadhesive was developed by Schreader et al. for bone-to-bone fixation. This material consisted of a foam-like bioadhesive containing 4,4-methylene diphenyl diisocyanate (MDI) and caprolactone-based diol (polyol) reinforced with hydroxyapatite nanoparticles [60]. The crosslinking occurred via moisture-curing polyurethane chemistry, which can influence the physical properties. However, the final physicochemical and functional properties were dependent on the chemistry and structure of polyol. This bioadhesive demonstrated strong bone-to-bone bonding with an adhesion strength of 4.47 MPa after 20 h, which is four-fold greater than conventional PMMA-based bone cement.

Several studies have focused on the development of PMMA-based bioadhesives for bone repair applications. These bioadhesives have been predominantly used in dentistry and orthodontics due to the weak adhesion to bone, especially in a wet environment. Another issue is the exothermal reaction that occurs during the polymerisation that can lead to cellular death and bone tissue necrosis. Enhancement of the adhesive strength of PMMA-based bioadhesives has been reported by enriching the adhesive with hydroxyapatite particles. However, despite the increase in adhesion strength, the lack of biodegradability has limited the clinical application as a bioadhesive for bone repair applications [130]. A bioadhesive that shows improvements in adhesive properties, particularly in an environment with high humidity, as well as improved biodegradation, have been achieved by Wistlich et al. [131]. They developed a bioadhesive for bone repair applications using a photocurable poly(ethylene glycol) dimethacrylate (PEGDMA) matrix, adding an isocyanate functional (six-armed) star-shaped prepolymer with ethylene oxide and propylene oxide copolymerised (NCO-sP(EO-stat-PO)) in a ratio of 4:1. The NCO-sP(EO-stat-PO enhanced the biodegradation properties and demonstrated a low level of cytotoxicity. Furthermore, the improved adhesive properties were achieved by modifying the matrix PEGDMA with biodegradable ceramic adjuvants (e.g., struvite (MgNH_4_PO_4_·6H_2_O), newberyite (MgHPO_4_·3H_2_O) or gypsum (CFaSO_4_·2H_2_O). In addition to improving the adhesive properties of the bioadhesive, these ceramic-based adjuvants also increased the porosity of the adhesive, leading to ingrowth of new bone via ion release. This bioadhesive has also been shown to be cytocompatible, easy to apply and demonstrate appropriate bone-to-bone adhesion in a wet environment, as well as supporting bone formation during fracture healing.

Fibrin-based natural polymers have also been applied clinically as bone adhesives, providing biocompatibility, biodegradability and cost effectiveness. These bioadhesives have been extensively used in bone tissue engineering applications, mainly for the acceleration, union and revascularisation of the osteochondral fragments [132,133]. An in vivo study demonstrated the formation of a dense network of osteoid tissue around tricalcium phosphate particles. Le Nihouannen et al. developed a bioadhesive by incorporating macro- and micro-porous biphasic calcium phosphate (MBCP) ceramic granules within a fibrin-based sealant (i.e., *Tissucol^®^*) [134]. In particular, 60% hydroxyapatite and 40% beta-tricalcium phosphate (β-TCP) were incorporated into the fibrin-based sealant and the osteoinductive properties evaluated. The formation of a well mineralised ectopic bone was observed between the MBCP particles, proving the ability of the MBCP-fibrin-based sealant to promote osteogenesis. Cassaro et al. developed a bioadhesive that included a fibrin-based biopolymer, which demonstrated haemostatic, sealant, adhesive, scaffolding and drug-delivery properties, and biphasic calcium phosphate (BCP) particles and mesenchymal stem cells (MSCs) [135]. Cassaro et al. demonstrated the bioadhesive to be cost-effective to manufacture, offering good biocompatibility as well as effective repair of the fractured bone and the formation of new bone. 

Polysaccharide-based bioadhesives have also been developed for bone repair applications. For instance, Kumbar et al. [136] investigated bioadhesives from cellulose derivatives such as cellulose acetate and ethyl cellulose, which are linear polysaccharides of D-glucose units linked by β(1→4) glycosidic bonds. The hydrogen-bonded structure resulting from the β(1→4) glycosidic bonds led to good biocompatibility and high mechanical properties. This study reported that the polysaccharide-based bioadhesive can form adhesive bonds between cellulose and bone through the carboxylic acid groups, as well as demonstrate a compressive strength (27–33 MPa) close to human trabecular bone. Two component bioadhesives derived from polysaccharides were developed by combining biocompatible chitosan or dextran with degradable starch [137] Initially, the polysaccharides were oxidised with periodic acid (L-3,4-dihydroxy-l-phenylalanine (DOPA)) to generate aldehyde groups, which is the main component found in mussels to help them adhere to the surface of a rock. In this bioadhesive, a covalent bond that is developed enabled a strong adhesion bond at the bone–bone interface as well as a high cohesion strength within the bioadhesive. This bioadhesive demonstrated excellent biocompatibility, with higher mechanical properties than fibrin glues.

L-DOPA, a hydroxylated form of tyrosine, has also been incorporated with the functional binder (mussel-derived adhesive protein (MAP)) to effectively retain deproteinised bovine bone mineral (DBBM) within the bone defect for bone tissue engineering applications [138]. Assessment of the biomechanical properties demonstrated the formation of an aggregate by the binding of the DBBM particles. An improvement in osteoconductivity and acquisition of osteoinductivity was observed, which resulted in an acceleration in bone remodelling and regeneration, with the density of new bone being similar to the normal bone. 

Sandcastle worm-based adhesives have shown particular promise in bone repair applications due to the ability to achieve rapid high strength adhesion in a wet environment. One such example is a water-borne adhesive modelled on the proteins from the sandcastle worm-based adhesive which was developed via the incorporation of phosphate, primary amine and catechol sidechains [127]. In particular, polymerised monoacryloxyethyl phosphate (MAEP), dopamine methacrylamide (DMA), acrylamide (Aam) and fluoroscein isothiocyanate (FITC)-methacrylamide were mixed together and applied to bond and stabilise bone fragments. The resultant bioadhesive demonstrated an adhesive strength 40% higher than cyanoacrylate-based bioadhesives. In vitro data demonstrated the ability of the sandcastle worm-based bioadhesive to bond bone fragments back together in a wet environment, while also exhibiting good biocompatibility and osteoconductivity. 

Gall et al. [139] developed a sandcastle worm-derived bioadhesive comprised of O-phospho-L-serine, a component of many proteins that exist in natural secretions, resulting in the development of a biodegradable bioadhesive that demonstrates almost instantaneous adhesion (≤10 s). O-phospho-L-serine is a phosphor-related amino acid component of osteopontin (OPN), which has a similar sequence to peptides of adhesion proteins and, when combined with calcium phosphates, leads to the development of a bioadhesive with high biodegradability and mechanical strength (i.e., adhesive and cohesive strength) within a short period [140]. An alternative approach by Kirillova et al., consisting of O-phosphoserine and tetracalcium phosphate, led to the development of another bioadhesive which exhibited a setting time of less than 10 min and the ability to achieve high bone-to-bone adhesive strength [124]. This bioadhesive demonstrated a shear strength ten-fold higher than calcium phosphate cements and PMMA bone cements. In addition to the high adhesive strength achieved, both sandcastle worm-derived bioadhesives also demonstrated osteointegration, bone ingrowth and biodegradability. 

Pujari-Palmer et al. reported a new class of sandcastle worm-derived calcium phosphate-based bioadhesives that have the potential to bond hard/soft tissue together and bond hard/soft tissue to metallic and polymeric prosthetic implants [125]. These marine-derived bioadhesives combined alpha tricalcium phosphate powder modified with phosphoserine. Phosphoserine is predominantly found in phosphoproteins that are involved in a range of biological processes, from adhesion, in marine-based bioadhesives, tissue adhesion, cohesion and load dissipation in animals, to biomineralisation, via matrix proteins and matrix vesicles. Pujari-Palmer et al. reported that phosphoserine can create an amorphous stable bioadhesive within a wet environment, improving the physicochemical properties, since they exhibited atomic-scale and macroscale interactions [125]. Furthermore, they reported that the existence of phosphoserine within the bioadhesive can lead to accelerated bone regeneration without causing any inflammation or adverse responses. A further study reported that these bioadhesives demonstrated adhesive strength when cured in wet-field conditions of 2.5–4 MPa (40-fold higher than commercial cyanoacrylates (0.1 MPa) and 100-fold higher when compared to surgical fibrin glue (0.04 MPa)) [141]. These bioadhesives have been shown to be effective in terms of the efficacy in bonding soft tissue (i.e., skin) ex vivo [132]. The bioadhesive provided a bond strength of 200 kPa within 30 min, while, after 90 min, the bond strength was close 332 kPa. The bond strength of the phosphoserine-based bioadhesive was 44-fold higher than for fibrin-based bioadhesive and 3-fold higher than mussel-derived bioadhesives. The assessment of the biodegradation behaviour of phosphoserine-based bioadhesives in physiological fluid ex vivo demonstrated the decrease of degradation increasing the density (lower porosity) and the surface area of the adhesive [132]. For bone tissue engineering applications, an effective bioadhesive requires high mechanical strength, low biodegradation and retention of bond strength within the initial days and weeks post-fracture stabilisation. The phosphoserine-based bioadhesive demonstrated a relatively high bond strength (39–50 MPa) and slow biodegradation (8–14% mass loss after 14 days) until the formation of new hard tissue, while also presenting amorphous calcium phosphate and metastable alpha-tricalcium phosphate on the surface of the bioadhesive [140]. Hulsart-Billström et al. [133] demonstrated the first in vivo biological safety assessment of a different phosphoserine-based bioadhesive for bone tissue engineering applications. The study demonstrated that all phosphoserine-based bioadhesives investigated supported a rate of cell proliferation of 45–64%, with no evidence of redness, swelling, inflammation, fibrotic tissue, disruption or bleeding. The lack of increased immune response and absence of ectopic bone formation demonstrated in this study confirms the highly desirable characteristics of sandcastle worm adhesives in order to achieve effective gluing of bone fragments while successfully guiding osteogenesis to promote bone repair and regeneration. 

### Reinforced Bioactive Adhesives for Bone Fracture Repair

**Figure 12:**
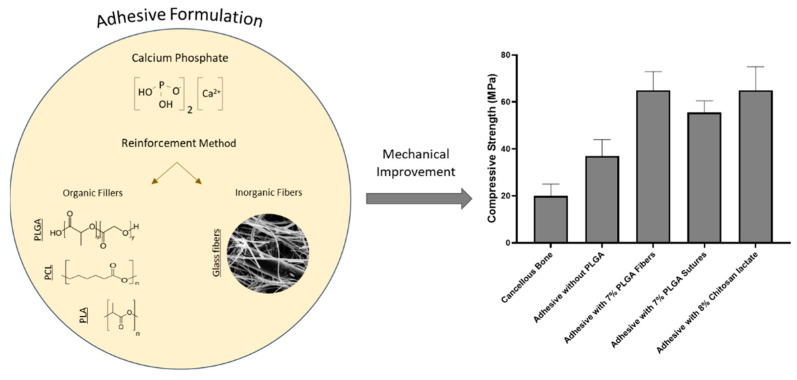
Improved mechanical properties can be achieved by incorporating organic (e.g., poly(lactic acid) (PLA), poly(glycolic acid) (PGA), poly(lactic-co-glycolic acid) (PLGA) and PCL)) and/or inorganic additives (e.g., glass fibres) to adhesives, thereby making them suitable to treat long bone fractures of the extremities.

In recent years, bioadhesives offering improved mechanical properties that can provide effective and faster bone fracture healing (e.g., osseointegration or stable microenvironment, osteoinduction and osteoconduction) have been developed. Improvement in mechanical properties has been achieved by the incorporation of various organic and inorganic additives (Figure 12). For instance, a mussel inspired adhesive was developed containing tetracalcium phosphate (TTCP) which contained PLGA fibres, leading to a biodegradable bone adhesive with excellent osseointegration properties [142]. The incorporation of 7 wt.% PLGA fibres exhibited a compressive strength of 62 ± 8 MPa and shear strength of 3.5 ± 0.6 MPa, which resulted in a two-fold increase when compared to the bioadhesive without PLGA fibres, along with improvement in stability of shape on setting, rapid setting time in wet environment, as well as excellent bioresorbability and osteoconductivity. An alternative reinforcement strategy using different wt.% of chitosan lactate solution [143] instead of pure water has also been investigated, which resulted in finer and more homogeneously dispersed pores within the microstructure of the adhesive and, as a consequence, improved mechanical properties. Even though using chitosan lactate as the liquid component in the adhesive offered good biocompatibility, biodegradability and osteoconductivity, it provided insufficient elasticity. Furthermore, a non-degradable biocompatible bone plate composed of nanohydroxyapatite/polyamide 66/glass fibre (n-HA/PA66/GF) has been developed to support the repair of loading-bearing bone fractures [144,145]. Histological analysis demonstrated good bone growth at the interface and integration of the plate with the native bone tissue. Ahlfeld et al. [146] developed a 3D printed implant comprising of fibrin gel and CPC. The fibrin gel was used as a highly degradable cell delivery system that enabled cell migration and, as a consequence, demonstrated excellent bone formation properties after 12 weeks.

For improvement of the osseointegration properties and the microenvironment stability, enhancement of the chemical and physical bonding (such as covalent, hydrogen and ionic bonds) is required. Enhancement of hydrogen bonding has been achieved by incorporating a supramolecular hydrogel network [147,148] which provided improvement of the interfacial toughness between disparate substrates and additional functionality, such as reversibility and self-healable adhesion. Liu et al. [149] incorporated starch and BaSO_4_ into CPC, achieving higher biodegradability and osteogenic properties. It also demonstrated injectability and setting time within the clinical requirements for minimally invasive bone repair applications.

**Figure 13:**
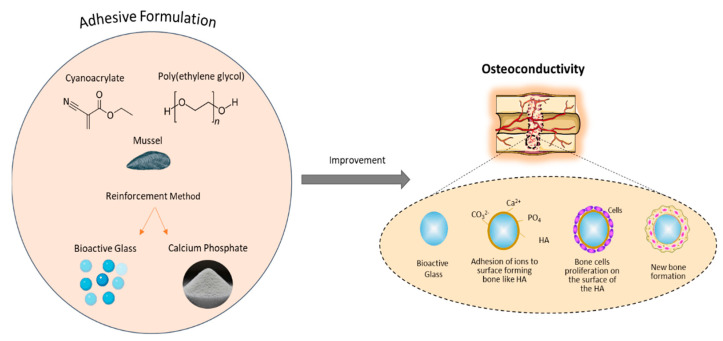
Improved osteoconductivity can be achieved by incorporating bioactive glasses and calcium phosphate-based materials into the adhesive, thereby providing space for bone cell migration, proliferation and differentiation.

Promotion of osteoblast growth on the bone surface is also advantageous for bone adhesives. Bioactive glasses and calcium phosphate material have been combined with adhesives to help promote bone tissue regeneration (Figure 13). A class of bioactive pore forming adhesive was developed by incorporating PEG porogens with encapsulated bioactive glass in 2-octyl cyanoacrylate (OCA) [57]. The reinforced adhesive exhibited accelerated HA formation ability and excellent bioactivity under physiological conditions, with superior mechanical properties, instant bonding and a high efficiency in terms of bone regeneration. Poly propylene fumarate (PPF)-based adhesives have been enhanced through the incorporation of bioactive glass nanoparticles [58]. Improvements regarding in vitro bioactivity, biodegradability, biocompatibility, bone adhesion and high cell viability demonstrated its potential as a biodegradable adhesive for use during orthopaedic surgery. Due to the excellent osteoconductivity of ceramic-based materials (e.g., HA, calcium carbonate and tricalcium phosphates) and their mechanical reinforcement potential, they have been used widely in bone fracture repair. Serano et al. evaluated the reinforcement of a chitosan-based adhesives using HA and calcium carbonate particles. The addition of the HA and calcium carbonate provided superior adhesive properties in both dry and aqueous conditions, combined with normal cell growth and excellent biocompatibility in vitro. Thiol-ene adhesives have been also modified by introducing HA, which improved the biocompatibility and in vivo functionality in terms of no cytotoxicity or genotoxicity, no inflammatory response, as well as no adverse effects on bone healing [150]. A polyurethane-based adhesive with HA nanoparticles was developed by Schreader et al. [60]. Increased adhesion was demonstrated compared to other conventional adhesives, and biocompatibility was confirmed through in vitro and preliminary in vivo analysis. However, long-term observations and additional tests are needed to demonstrate full in vivo efficacy. Erken et al. [151] and Lie et al. [152] developed polyurethane-based adhesives with β-TCP, which demonstrated enhanced mechanical properties and the ability to facilitate osteoconduction. Bioinspired mineral–organic bone adhesives comprised of tannic acid (TA), silk fibroin (SF) and HA have also been reported, with acceleration of bone regeneration (in vivo) and closure of fracture having been observed [153].

**Figure 14:**
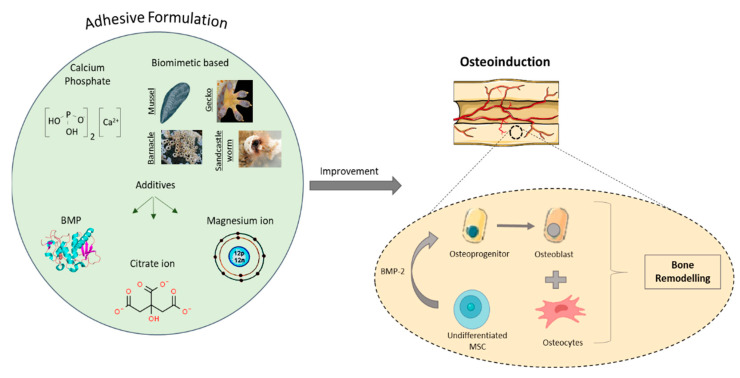
Improved osteoinduction can be achieved by incorporating certain growth factors, organic compounds and elements into the adhesive, thereby promoting the differentiation of undifferentiated cells into osteoblasts.

Improvement of the osteoinductive potential of bone adhesives has been investigated (Figure 14). Bai et al. [153] introduced BMP-2 into an adhesive containing SF, TA and HA to promote osteoinductivity. The differentiation of mesenchymal stem cells (MSCs) into osteoblasts, by the expression of alkaline phosphatase (ALP), was demonstrated. BMP-2 absorption onto β-TCP was evaluated for its use as a delivery vector for bone regeneration [154]. The results indicated that local administration of BMP-2/β-TCP in the tooth extraction socket significantly induced bone formation and reduced bone necrosis, with direct regulation of osteoblast differentiation and osteoclast activity achieved due to the BMP-2. In addition to BMP, citrate ions have been studied, since they can be consumed by MSCs to increase osteogenesis. Due to this mechanism, the design of a citrate-based adhesive was explored [155]. Ma et al. [156] developed a biomimetic citrate-based adhesive that acted as an osteopromotive factor and supported osteogenic differentiation. Similar studies by Xie et al. demonstrated that the citrate-based mussel-inspired bioadhesive was highly injectable and, when evaluated in vivo using a rabbit fracture model, promoted organised bone formation with markedly enhanced mechanical properties [157]. Magnesium ions promote osteogenic differentiation of the encapsulated hMSCs and ALP activation [158]. Various studies have investigated the incorporation of magnesium ions into conventional adhesives, leading to improvements in inducing osteoblast differentiation and faster healing [159]. However, to date, their poor degradability has limited their clinical translation for the treatment of bone fracture repair [160].

## 7. Conclusions and Future Research Directions

Considering the disadvantages of existing surgical approaches for the treatment of complex bone fractures, bioadhesives for bone tissue engineering applications present significant potential as an alternative minimally invasive surgical approach. One main challenge relating to the development of bioadhesives is the requirement to achieve high bond strength within the challenging clinical environment (i.e., wet environment). However, bioadhesives have the potential to offer advantageous properties, including biocompatibility, biodegradation/bioresorbability, osteoconductivity and high bond strength to hard tissue (i.e., bone), and, to date, a range of such bioadhesives has been investigated, including synthetic-polymer-, biological-polymer- and biomimetic-based adhesives. Many studies have focused on the development of bioadhesives with the ability to provide a high bond strength within a wet environment, while at the same time combining the requirement for biocompatibility and biodegradability. Despite these challenges, a number of promising approaches, such as polysaccharide- or protein-based bioadhesives that achieve high levels of adhesion through covalently bonding to hard/soft tissue, are currently at the early stages of clinical testing. However, these bioadhesives are not suitable for application within a wet environment, which presents a significant limitation for clinical use. Moreover, despite the high adhesive strength, they require photoirradiation, which has a detrimental effect on the neighbouring healthy tissue. 

Biomimetic-based bioadhesives that have been inspired by examples of adhesion found in nature present an attractive alternative approach and are rapidly gaining momentum in the field of biologically applicable bioadhesives. They offer a significant advantage as they can function in a wet environment. Currently, the scientific knowledge and understanding of the design rules associated with underwater adhesion is limited, and considerable research efforts are being invested into the study of adhesion in living systems. With more substantial and exhaustive investigation relating to the interplay of environmental and chemical/biological factors, chemistries and mechanisms for effective natural adhesion, it has been demonstrated that biomimetic-based bioadhesives have a potential role to play in effective stabilisation and repair in bone tissue engineering applications, including the treatment of complex bone fractures. Comparing these biomimetic-based bioadhesives, systems that mimic the sandcastle worm are considered the most promising. For instance, the sandcastle worm-inspired bioadhesive that uses the addition of a phosphorylated amino acid (e.g., phosphoserine) to calcium phosphate-based adhesives can be considered as a highly effective bone adhesive for bone fracture stabilisation and repair. Phosphoserine can create novel properties in bioceramics, such as high adhesion within a few seconds and a reduction in the inherent brittleness displayed by bioceramic materials. 

Research development of the bioadhesives is focused on exploring their potential as a vehicle for the controlled and localised delivery of cells, growth factors and small molecules [161,162,163], focusing on the synchronisation of the load and release of these bioactive elements with the timeline of normal tissue healing/repair. Another area of research focus relates to tuning the in vivo biodegradation of bioadhesives, which would complement localised delivery of a particular cargo [163]. In addition, studies have been focused on the demonstration of a non-toxic, biocompatible, biodegradable adhesive that can be easily delivered using minimally invasive surgical approaches for on-demand and precise mixing/delivery and that can be manufactured at scale [124,125,127]. Considerable research remains on improving the suboptimal mechanical and physical properties (e.g., adhesion strength, bulk modulus, injectability and ultimate strain prior to breakdown for the functionality of repaired tissue) and biocompatibility for cell support and tissue ingrowth with minimal cytotoxicity, particularly under wet conditions.

To conclude, bioadhesives have gained increasing significance in recent years, since they demonstrate potential as adhesives for fracture repair, bone filling and augmentation for bone implants, and they have demonstrated the ability to promote tissue repair and bone formation due to the ability to release essential bioactive cues [164]. Despite the challenges related to achieving a material that can combine optimal physical and bio/chemical properties with biocompatible and non-toxic behaviour, it is anticipated that the ongoing research developed in this area will provide clinically applicable bioadhesives with an enormous potential to promote musculoskeletal repair and regeneration. 

## Figures and Tables

**Table 1:** Comparison of the different properties of all the synthetic-based adhesives.

Scheme			
	**Application**	**Advantages**	**Disadvantages**
Cyanoacrylates [45,49,50,51]	Craniofacial, osteochondral and trabecular fractures Bone formation and fragments fixation Enhancement or replacement of screws/plates	Max adhesive strength of 9 MPa Enhanced tensile and shear bond in wet and dry environment Higher shear strength (1–2 MPa) than screws and plates	Partial bone formation Less efficient than screws with low adhesive and mechanical properties Chronic inflammatory response and tissue necrosis Cytotoxicity to cells in vitro and dermatitis in vivo
Polyurethane [53,54,55,56]	Bone formation and fragments fixation Bone to bone adhesion Closure of fractures	High adhesive or/and cohesive strength Osteogenic, non-toxic and biocompatible Degradation in wet environment	Bond failure between bone and adhesive Low biodegradability Infection Tissue necrosis
Polyester [58,59,69]	Scaffold in bone regeneration Tissue adhesion	Faster degradation in wet environment than polyurethane-based High mechanical & adhesion strength	Mechanical stability during degradation Osteogenic capacities (osteoconduction and osteoinduction) Inflammation at the application site Low yield strength Significant cytotoxicity
Poly-methyl methacrylate (PMMA) [70,71]	Bone fragment and implant fixation Adhesives in dentistry Bone formation	Hydrophobic behaviour Increased bonding to wet bone Easy application Cytocompatibility	Low adhesive strength Thermal necrosis of bone tissue Lack of biodegradability
